# Oxidative damage, antioxidant mechanism and gene expression in tomato responding to salinity stress under *in vitro* conditions and application of iron and zinc oxide nanoparticles on callus induction and plant regeneration

**DOI:** 10.1186/s12870-021-03379-7

**Published:** 2021-12-16

**Authors:** Mohammad Ali Aazami, Farzad Rasouli, Asghar Ebrahimzadeh

**Affiliations:** Department of Horticulture, Faculty of Agriculture, University of Maragheh, Maragheh, Iran

**Keywords:** Antioxidant defense genes, *In vitro*, Nanoparticle, Salinity, *Solanum Lycopersicon* L

## Abstract

**Background:**

Salinity is one of the most challenging abiotic stresses restricting the growth of plants. *In vitro* screening will increase the efficiency and speed of salinity tolerant genotypes identifications. The response of four tomato cultivars under salinity was analyzed *in vitro* to evaluate the seedlings growth, biochemical, and gene expression responses as well as the effect of nano zinc and iron on callus induction and plant regeneration.

**Results:**

The results showed that an increase in salinity stress in the medium decreased the germination percentage, fresh and dry weight of shoot, root length, chlorophyll a, b and carotenoids content, K and Ca content, and on the other hand, Na content was increased. MDA content (‘Nora’, ‘PS-10’, ‘Peto’ and ‘Roma’: 1.71, 1.78, 1.66 and 2.16 folds, respectively), electrolyte leakage (‘PS-10’: 33.33%; ‘Roma’: 56.33%), were increased with salinity of 100 mM compared to control. Proline content was increased in 50 mM NaCl (10.8 fold). The most activity of antioxidant enzymes including CAT, SOD, APX, GPX, and GR was observed in the ‘PS-10’ cultivar, and the lowest activity of these enzymes was observed in ‘Roma’ under salinity stress. The AsA and GSH were decreased and DHA and GSSG were increased with the increased intensity of salinity. The relative expression of *SOD*, *APX*, and *GR* genes varied in different cultivars at different salinity concentrations. The most percentage of callus induction was observed with applying iron oxide nanoparticles, and the most regeneration rate was recorded using zinc oxide nanoparticles.

**Conclusion:**

The results showed that salt-tolerant cultivars such as ‘PS-10’ with better osmotic adjustment, are suitable candidates for the future production and breeding programs. The use of nutrient nanoparticles under salinity stress for different tomato cultivars increased their performance.

## Background

Tomato (*Solanum lycopersicon* L.) is an economically important crop cultivated worldwide. The majority of plants, especially tomato, are sensitive to abiotic stresses; although the responses are different between cultivars [1]. Hence, plants screening and breeding are important to analyze the tolerance against salinity stress impacts [2, 3].

The intensification of various abiotic stresses has been changed into a major threat to the sustainable production of agricultural crops [4–6]. Plants show physiological, biochemical, and genetic responses to salinity stress [7, 8]. Because of its ability to compete with essential materials, NaCl is one of the most vital factors of soil salinity, which leads to a lack of several nutrients in the plants, and causes toxicity of plants [9]. At the same time, countless destructive effects through the congestion of reactive oxygen species (ROS) can result in oxidative stress [4–6, 10]. Salinity stress is created by various ions, especially Na and Cl, which can be transferred to cells and inside cell organelles. Salinity stress can also leave a special effect on the ions movement and accumulation in the environment [3, 11]. Researchers have reported that the activity of antioxidants and antioxidant enzymes varies due to the salinity level, the exposition time and the growing stage of plants [12]. Reactive oxygen species (ROSs) can be considered not only as a cellular signal of stresses, but they also are secondary messengers involved in signaling routes of stress responses. Over-production of ROS in peroxisomes and chloroplasts is correlated to a strong change in gene expression [13]. Gene expression of antioxidant enzymes under salinity and drought stress were examined in multiple studies. In a study, ascorbate peroxidase (*APX)* gene expression was increased under drought conditions, however, there was no change in *SOD* and *CAT* gene expression [14, 15]. The results showed differential expression of *SOD*, *CAT*, and *APX*, which is irrelevant to changes in their enzyme activity. The majority of genes play a vital role in plant response to stresses, and improvement of stress tolerance. Along with many antioxidant enzyme genes (*SODs*, *CATs*, and *APXs*), over-ectopic expression of dehydration-responsive element-binding (DREBs) factors in plants can increase stress tolerance [15, 16].

*In vitro* techniques pave the way for screening a number of required genotypes for stress tolerance in different growing and breeding steps [17]. In most species such as mulberry, grapes, pistachio, tomato, citrus fruits, and carrots, the salinity-tolerant lines have been separated using *in vitro* techniques [18–20].

Nanotechnology is an emerging technology, and the development of nanomachines and nanomaterials has opened new applications in plant and agricultural biotechnology [21]. Successful use of nanoplatforms *in vitro* has created interest in nanotechnology in agriculture. The application of nanomaterials can help the faster plant germination; improvement of production through creating tolerance against biotic and abiotic stresses; optimal use of nutrients, and increased growth of plants with reducing environmental effects compared to traditional approaches [22, 23]. Reynolds [24] showed that the nanoparticle (NP) nutrients can be used for production purposes and enhancement of performance. It seems that the signaling of nano zinc oxide (ZnO), and iron oxide plays a key role in the regulation of various mechanisms in response to abiotic stresses in plants [25]. It has been specified that zinc and iron play crucial roles in the management of reactive oxygen species (ROS) and the protection of plant cells against oxidative stresses [25–27]. Prasad et al. [28] have studied the effect of ZnO in nanoscale on germination, growth, and yield of peanut, and observed higher growth and yield. Various studies are available for the interaction of salinity and ZnO in higher plants. These NPs in their oxide form for example; ZnO, Fe_2_O_3_ and SiO_2_ are reported to enhance the germination, growth, vitality and biochemical parameters of plants [29].

Because of intense salinity effects, and economic importance of tomatoes, as well as the increasing use of tissue culture methods for screening to stresses and producing crops, the present study has been conducted to analyze the effects of salinity stress on seedling growth, biochemical properties and gene expressions related to antioxidant enzymes in tomato cultivars. Also, the experiment has elucidated the decreased harmful effects of salinity *in vitro* using ZnO and Fe_3_O_4_ on callus induction, and regeneration of shoots in four commercial tomato cultivars.

## Results

### The effect of salinity on seedling properties

With increased salinity stress under *in vitro*, the germination of tomato seeds was decreased in all cultivars. The germination percentage in ‘PS-10’, ‘Peto’, ‘Nora’ and ‘Roma’ cultivars was decreased from control to NaCl (100 mM) and from 100 to 41.67%, 95 to 15% 80 to 10% and 83.33 to 8.33%, respectively. An increase in salinity caused a decrease in the fresh and dry weight of the shoot. The highest fresh and dry weight of shoots respectively with 338 and 89.33 mg (control) was relevant to the ‘PS-10’ cultivar. Also, the highest fresh and dry weight in the salinity level of 100 mM of NaCl was 160.5 and 55.73 mg in ‘Peto’ cultivar. The shoot length was decreased with increasing salinity stress *in vitro* in all cultivars. ‘PS-10’ with a shoot length of 9.30 cm in 100 mM NaCl showed the most shoot length compared to other cultivars. The fresh and dry root weight was decreased in higher salinity. The highest fresh and dry root weight was relevant to ‘PS-10’, respectively with 101.8 mg (control), and 59.15 (100 mM of NaCl), and 32.13 (control), and 21.30 (100 mM of NaCl) (Table 1). The root length in all cultivars was decreased *in vitro* under salinity stress.

**Table 1:** The effects of NaCl treatment on germination traits of four tomato cultivars in *in vitro* condition. Means followed by the same letter on columns are not significantly different at 0.05 level, according to the Duncan’s multiple range test. Data are mean ± SD (*n* = 4 replicates)

Character	Concentration NaCl (mM)	Genotype			
		‘Nora’	‘PS-10’	‘Peto’	‘Roma’
Germination (%)	0	80.00 ± 4.71^c^	100.0 ± 0^a^	95.00 ± 2.35^ab^	83.33 ± 2.72^c^
	25	43.33 ± 2.72^e^	88.33 ± 1.36^bc^	90.00 ± 4.71^a-c^	56.67 ± 7.2^d^
	50	23.33 ± 2.72^fg^	81.67 ± 1.36^c^	55.00 ± 2.35^d^	23.33 ± 2.72^fg^
	75	16.67 ± 2.72^gh^	63.33 ± 2.72^d^	33.33 ± 2.72^ef^	13.33 ± 2.72^gh^
	100	10.00 ± 0^h^	41.67 ± 1.36^e^	15.00 ± 2.35^gh^	8.33 ± 1.36^h^
Shoot wet weight (mg)	0	303.9 ± 0.88^b^	338.0 ± 4.56^a^	336.4 ± 4.47^a^	242.3 ± 3.98^d^
	25	207.4 ± 10.39^e^	288.8 ± 1.43^b^	267.3 ± 1.89 ± ^c^	179.3 ± 2.03^f^
	50	180.4 ± 3.55^f^	226.2 ± 0.92^d^	208.1 ± 0.83^e^	132.3 ± 1.16^h^
	75	143.5 ± 1.69^gh^	178.6 ± 1.93^f^	181.9 ± 3.08^f^	104.6 ± 1.07^i^
	100	128.8 ± 0.22^h^	154.2 ± 0.99^g^	160.5 ± 2.86^g^	80.98 ± 1.02^j^
Shoot dry weight (mg)	0	80.47 ± 0.45^b^	89.33 ± ^0.41a^	87.57 ± 0.47^a^	66.37 ± 0.69^e^
	25	66.43 ± 0.53^e^	80.73 ± 0.59^b^	75.00 ± 0.69^c^	52.67 ± 0.83^i^
	50	56.20 ± 0.91^h^	69.03 ± 0.46^d^	64.20 ± 0.46^f^	41.40 ± 0.33^l^
	75	49.30 ± 0.32^j^	59.17 ± 0.21^g^	60.57 ± 0.62^g^	36.00 ± 0.51^m^
	100	45.57 ± 0.28^k^	54.50 ± 0.38^hi^	55.73 ± 0.95^h^	30.83 ± 0.36^n^
Shoot length (cm)	0	13.07 ± 0.11^c^	14.17 ± ^0.16ab^	14.37 ± 0.23^a^	11.47 ± 0.19^d^
	25	11.67 ± 0.21^d^	13.70 ± 0.18^b^	12.73 ± 0.16^c^	9.33 ± 0.11^g^
	50	9.90 ± 0.12^fg^	11.73 ± 0.16^d^	10.73 ± 0.16^e^	7.26 ± 0.15^j^
	75	8.20 ± 0.14^i^	10.10 ± 0.11^f^	9.30 ± 0.21^gh^	5.73 ± 0.26^k^
	100	7.50 ± 0.12^j^	9.30 ± 0.21^gh^	8.67 ± 0.19^hi^	5.50 ± 0.14^k^
Root wet weight (mg)	0	90.26 ± 0.55^d^	101.8 ± 2.21^a^	97.42 ± 1.65^ab^	77.41 ± 1.34^e^
	25	76.86 ± 1.81^e^	95.48 ± 1.37^bc^	91.38 ± 1.22^cd^	59.16 ± 0.75^h^
	50	55.77 ± 0.63^hi^	80.47 ± 0.99^e^	72.25 ± 1.35^f^	37.16 ± 0.94^k^
	75	46.80 ± 1.23^j^	65.78 ± 1.18^g^	59.83 ± 1.74^h^	29.66 ± 1.36^l^
	100	36.76 ± 0.76^k^	59.15 ± 1.27^h^	52.85 ± 0.79^i^	18.65 ± 0.52^m^
Root dry weight (mg)	0	28.80 ± 0.12^c^	32.13 ± 0.63^a^	30.70 ± 0.49^ab^	24.73 ± 0.39^e^
	25	25.33 ± 0.51^e^	31.13 ± 0.42^a^	29.33 ± 0.28^bc^	19.63 ± 0.23^h^
	50	19.23 ± 0.19^h^	27.13 ± 0.43^d^	24.47 ± 0.47^ef^	13.00 ± 0.38^j^
	75	16.73 ± 0.41^i^	23.13 ± 0.39^f^	21.07 ± 0.56^g^	10.63 ± 0.49^k^
	100	13.47 ± 0.25^j^	21.30 ± 0.41^g^	18.97 ± 0.31^h^	6.90 ± 0.18^l^

Mean with the same letter are not significantly different by Duncan grouping at (*P < 0.05*)

### Chlorophylls and carotenoids content

**Fig. 1:**
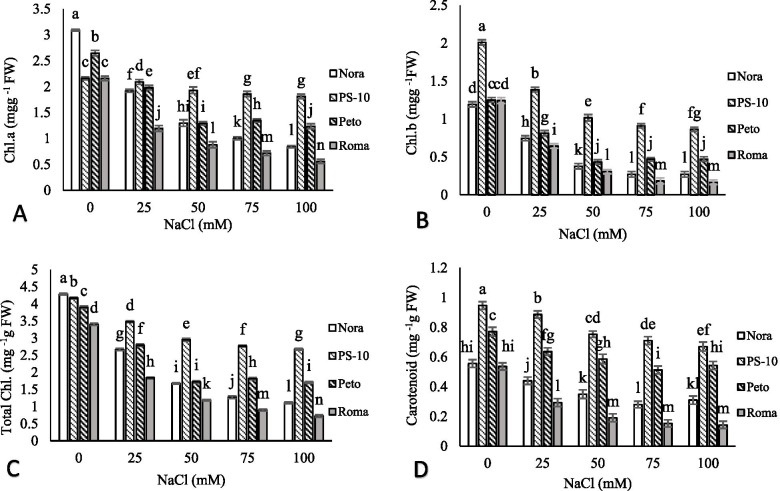
The effects of NaCl treatment on chlorophyll a (**A**), chlorophyll b (**B**), total chlorophyll (**C**) and carotenoid (**D**) content of four tomato cultivars in *in vitro* condition. Means followed by the same letter on columns are not significantly different at 0.05 level, according to the Duncan’s multiple range test. Data are mean ± SD (*n* = 4 replicates)

The highest content of chlorophyll a belonged to ‘Nora’ in the control, and the lowest content was observed in ‘Roma’ in 100 mM NaCl. Chlorophyll a content in ‘Nora’, ‘PS-10’, ‘Peto’ and ‘Roma’ cultivars decreased by 72.78, 16.2, 53.4% and 74.53% in 100 mM NaCl compared to the control, respectively. The highest content of chlorophyll b in the control with 2.01 mgg^−1^FW was observed in ‘PS-10’cultivar. Chlorophyll b in ‘Nora’, ‘PS-10’, ‘Peto’ and ‘Roma’ cultivars decreased by 77.36, 57.06, 62.4 and 86.64% in 100 mM NaCl compared to the control, respectively. The highest content of total chlorophyll was related to ‘Nora’ in the control. With increasing salinity stress, the content of total chlorophyll in all tomato cultivars decreased. The highest decrease in total chlorophyll was observed in ‘Roma’ cultivar with 78.75% and the lowest decrease in total chlorophyll was recorded in ‘PS-10′ with 35.89%. With increasing salinity stress, carotenoid content decreased in all tomato cultivars. The content of carotenoids in ‘Nora’, ‘PS-10′, ‘Peto’ and ‘Roma’ under 100 mM NaCl decreased by 44.24, 29.17, 29.48 and 73.32%, respectively, compared to the control. The highest reduction of carotenoids content was observed in ‘Roma’ after salinity increase from 50 to 100 mM (Fig. 1).

### Balance of elements

**Fig. 2:**
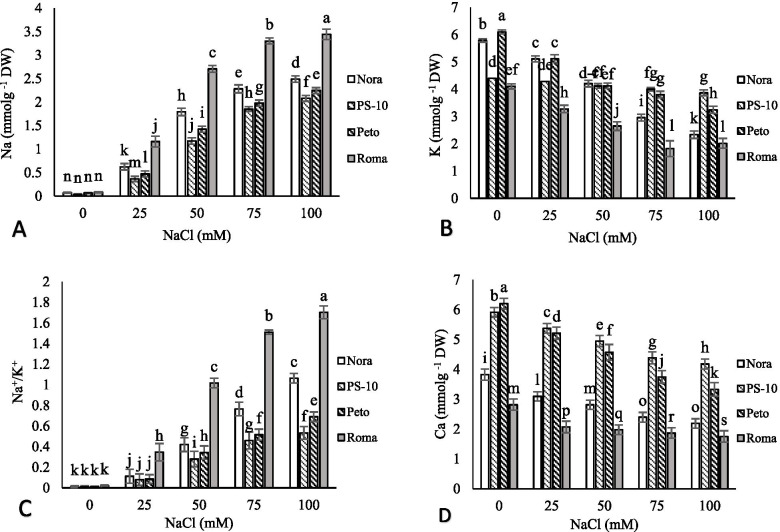
The effects of NaCl treatment on Na (**A**), K (**B**), Na/K (**C**) and Ca (**D**) content of four tomato cultivars in *in vitro* condition. Means followed by the same letter on columns are not significantly different at 0.05 level, according to the Duncan’s multiple range test. Data are mean ± SD (*n* = 4 replicates)

With an increase in salinity stress *in vitro*, the accumulation of sodium was increased in shoots of different cultivars. The highest accumulation of sodium was observed in shoots of ‘Roma’ cultivar (4.33 mmolg^−1^Dw) under 100 mM NaCl. In ‘Nora’, ‘PS-10’, ‘Peto’ and ‘Roma’ cultivars we observed 64.42, 42.22, 65.95 and 58.51% increase in sodium accumulation in 100 mM NaCl treatment compared to the control, respectively. With increasing salinity stress in culture medium, the content of potassium in all tomato cultivars decreased. The highest content of potassium was observed in ‘Peto’ in control and, the lowest content of potassium in ‘Roma’ cultivar was observed in 75 and 100 mM NaCl. In 100 mM NaCl, compared to the control; the highest decrease (59.68%) in potassium was observed in ‘Nora’ and the lowest decrease in potassium with 12.04% in ‘PS-10’. The highest Na^+^/K^+^ ratio was observed in 100 mM NaCl of Roma cultivar and the lowest Na^+^/K^+^ ratio was recorded in ‘PS-10’. With increasing salinity, calcium accumulation in shoots of all tomato cultivars decreased. The highest amount of calcium was observed in ‘Peto’ in control, and the least data for calcium content in Roma cultivar was observed in 100 mM NaCl. In ‘Nora’, ‘PS-10’, ‘Peto’ and ‘Roma’, calcium content decreased by 42.71, 29.3, 46.29 and 37.77% in 100 mM NaCl, compared to the control, respectively (Fig. 2).

### Osmolytes and membrane damage

**Fig. 3:**
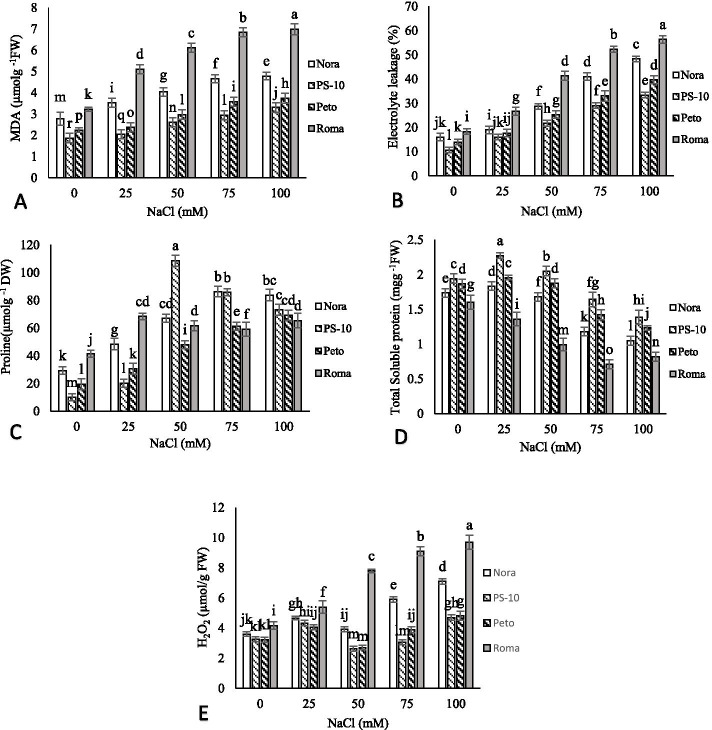
The effects of NaCl treatment on malondialdehyde (MDA) (**A**), Electrolyte leakage (EL) (**B**), proline (**C**), total soluble protein (**D**) and H_2_O_2_ (**E**) amount of four tomato cultivars in *in vitro* condition. Means followed by the same letter on columns are not significantly different at 0.05 level, according to the Duncan’s multiple range test. Data are mean ± SD (*n* = 4 replicates)

Malondialdehyde (MDA) content increased in all tomato cultivars with increasing salinity stress under *in vitro* conditions. In 100 mM NaCl, ‘Roma’ showed the highest content of MDA and PS-10 cultivar showed the lowest content of MDA. In ‘Nora’, ‘PS-10’, ‘Peto’ and ‘Roma’, the amount of MDA in 100 mM NaCl increased by 1.71, 1.78, 1.66 and 2.16 folds compared to control, respectively,. With increasing NaCl in the medium, membrane damage and ion leakage increased in all tomato cultivars. In 100 mM NaCl, ‘Roma’ with 56.33% showed the highest and ‘PS-10’ with 33.33% showed the lowest electrolyte leakage. The amount of H_2_O_2_ in 50 mM NaCl decreased in both ‘PS-10’ and ‘Peto’ cultivars compared to the control, and then raised with increasing salinity concentration *in vitro*. The highest amount of H_2_O_2_ was related to ‘Roma’ in 100 mM NaCl and the lowest amount was related to ‘PS-10’ in 50 mM NaCl. With increasing salinity stress, the content of proline in all cultivars increased. The highest and lowest proline levels were related to ‘PS-10’ in 50 mM NaCl and control, respectively. With increasing salinity stress up to 100 mM, proline content of ‘PS-10’ decreased. In ‘Nora’, ‘PS-10’, ‘Peto’ and ‘Roma’ cultivars, the content of proline in 100 mM NaCl increased by 3.43, 7.33, 3.58 and 3.51 fold, respectively, compared to the control. With increasing salinity stress, a relative decrease in total soluble protein was observed in all tomato cultivars. The highest content of total soluble protein in 25 mM NaCl was observed in ‘PS-10’ and the lowest content of total soluble protein in 75 mM NaCl was observed in ‘Roma’ cultivar (Fig. 3).

### Antioxidant enzymes activity

**Fig. 4:**
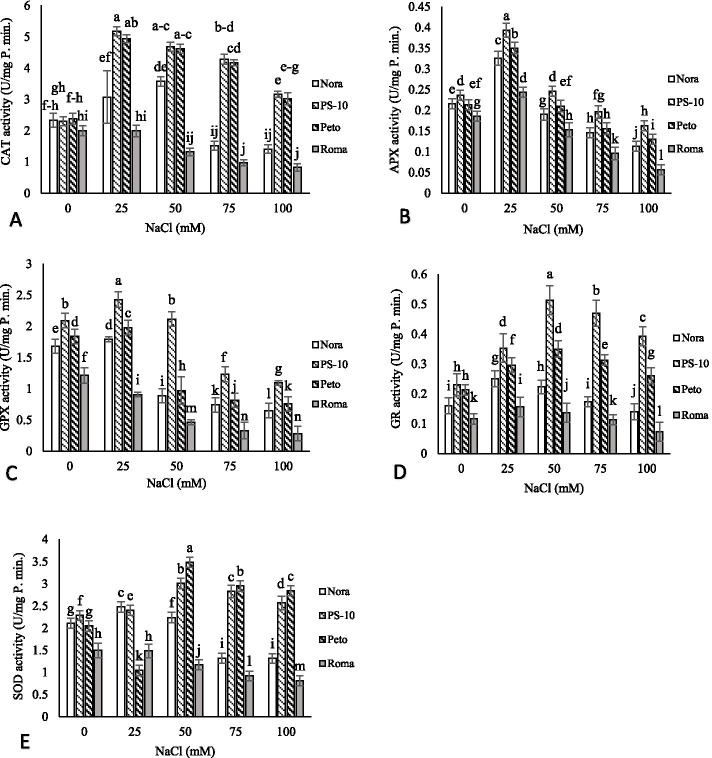
The effects of NaCl treatment on Catalase (CAT) (**A**), Ascorbate peroxidase (APX) (**B**), Guaiacol peroxidase (GPX) (**C**), Glutathione reductase (GR) (**D**) and Superoxide dismutase (SOD) (**E)** activity of four tomato cultivars in *in vitro* condition. Means followed by the same letter on columns are not significantly different at 0.05 level, according to the Duncan’s multiple range test. Data are mean ± SD (*n* = 4 replicates)

The activity of antioxidant enzymes varied in different tomato cultivars and with various salinity treatments. Moreover, the tolerant cultivars showed high antioxidant enzyme activity in different salinity levels. The CAT activity increased at all salinity levels in ‘PS-10’ and ‘Peto’ cultivars compared to the control. Catalase activity in ‘PS-10’ cultivar in 25 mM NaCl increased by 225.08% compared to the control. In ‘Roma’ cultivar, catalase activity in 100 mM NaCl decreased by 58.85% compared to the control, but no significant difference was observed between different salinity treatments in this cultivar. The highest APX activity was observed in all cultivars in 25 mM NaCl. ‘PS-10’ had the highest APX enzyme activity in 25 mM NaCl with 167.79% compared to the control. APX activity in ‘Roma’ decreased by 69.89% in 100 mM NaCl compared to the control. The activity of GPX enzyme in 25 mM NaCl increased in all cultivars except ‘Roma’ compared to the control and then decreased with increasing salinity levels. The highest GPX activity in 25 mM NaCl compared to the control was observed in ‘PS-10’ (115.95%) and the lowest amount of GPX enzyme activity in ‘Roma’ cultivar was observed in 75 and 100 mM NaCl compared to the control (72.79 and 76.66%, respectively). The GR enzyme activity increased in both cultivars of ‘PS-10’ and ‘Peto’ at all salinity levels compared to the control. The activity of GR in ‘PS-10’ under 50 mM NaCl increased by 223.04% compared to the control and the activity of GR in cultivar Roma in 100 mM NaCl decreased by 37.06% compared to the control. The amount of SOD enzyme activity in ‘PS-10’ and ‘Peto’ increased in all salinity levels compared to the control The amount of SOD enzyme activity in ‘Peto’ cultivar in 50 mM NaCl increased by 170.09% compared to the control. In 100 mM NaCl treatment, compared to the control; SOD activity in ‘Nora’ and ‘Roma’ cultivars decreased by 37.67 and 45.69%, and in ‘PS-10’ and ‘Peto’ cultivars increased by 112.37 and 138.67%, respectively (Fig. 4).

### Antioxidant activity

**Fig. 5:**
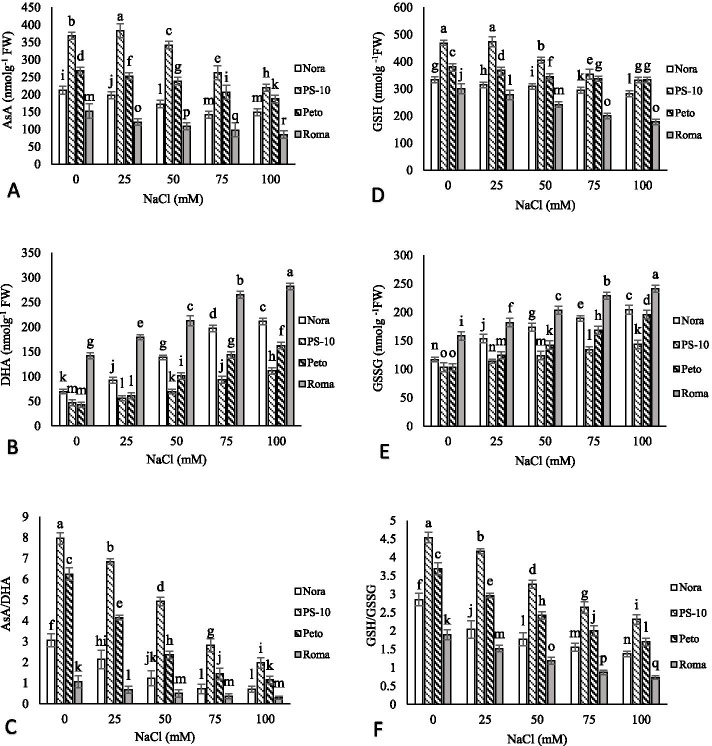
The effects of NaCl treatment on ascorbate (AsA) (**A**), Dehydroascorbate (DHA) (**B**), AsA/DHA (**C**), Reduced glutathione (GSH) (**D**), Oxidized glutathione (GSSG) (**E**) and GSH/GSSG (**F**) content of four tomato cultivars *in vitro* condition. Means followed by the same letter on columns are not significantly different at 0.05 level, according to the Duncan’s multiple range test. Data are mean ± SD (*n* = 4 replicates)

Ascorbate and AsA/DHA ratio decreased with increasing salinity levels. The highest content of AsA was observed in ‘PS-10’ at 25 mM NaCl. In other tomato cultivars, less AsA was observed in different levels of salinity stress than the control. As salinity increased, the content of dehydroascorbate (DHA) increased. The ‘Roma’ cultivar had the highest content of DHA compared to other cultivars at all treatment levels. At 100 mM salinity compared to the control in ‘Nora’, ‘PS-10’, ‘Peto’ and ‘Roma’ cultivars, AsA levels decreased by 29.81, 40.45, 30.19 and 44.40%, respectively. The highest AsA/DHA ratio was observed in ‘PS-10’ in control and, the lowest AsA/DHA ratio in ‘Roma’ cultivar was observed in 100 mM NaCl. GSH and GSH/GSSG decreased with increasing salinity stress. At 100 mM NaCl, compared to the control in ‘Nora’, ‘PS-10’, ‘Peto’ and ‘Roma’ cultivars, GSH levels decreased by 15.32, 29.14, 12.82 and 40.9%, respectively. Salinity stress caused a significant increase in GSSG in different tomato cultivars. The highest content of GSSG was related to ‘Roma’ in 100 mM NaCl. The lowest content of GSSG was observed in ‘Peto’. GSH/GSSG ratio with increasing NaCl levels showed a significant decrease in all tomato cultivars (Fig. 5).

### Gene expression

**Fig. 6:**
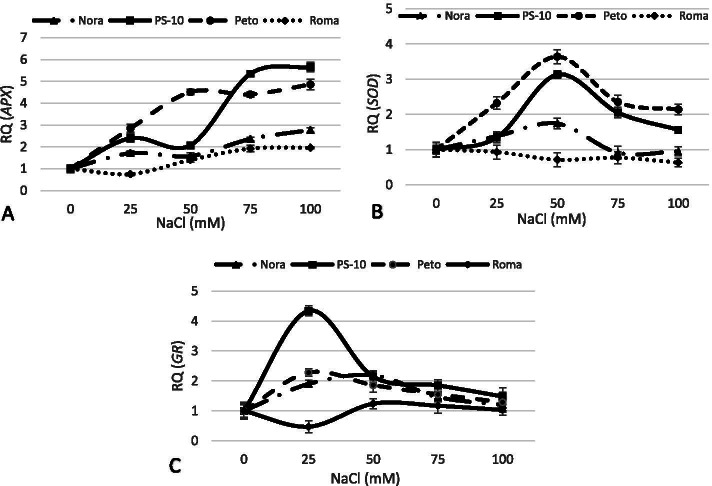
Quantitative expression analysis of *APX* (**A**)*, SOD* (**B**) and *GR* (**C**) genes in four tomato cultivars exposed to salinity *in vitro* condition. Data are mean ± SD (*n* = 4 replicates)

The relative expression of *SOD*, *APX*, and *GR* genes in four cultivars under various NaCl stress levels was determined using qRT-PCR. The qRT-PCR data were normalized using actin, a housekeeping gene. The relative expression of the *SOD* gene varied in different NaCl levels. The most *SOD* gene expression level in ‘Peto’ cultivar with 3.64-fold was relevant to 50 mM of NaCl. In ‘Peto’, ‘PS-10’, and ‘Nora’ cultivars, the relative *SOD* gene expression was increased with increasing salinity up to 50 mM and was then decreased in 75 and 100 mM. The most relative expression of *APX* gene up to salinity level of 50 mM NaCl was associated with ‘Peto’ cultivar with 4.51-fold. With salinity level up to 75 and 100 mM, the most level of relative *APX* gene expression was observed in ‘PS-10’ with 5.66-fold. The least relative *APX* gene expression in ‘PS-10’ cultivar was associated with the treatment of 50 mM NaCl (2.07-fold), and the gene expression was increased (2.58-fold) in 75 mM NaCl. The most relative *GR* gene expression in ‘PS-10’ cultivar was observed in 25 mM NaCl (4.34-fold). Then after, increased salinity level in this cultivar decreased the relative *GR* gene expression, so that the *GR* gene expression varied up to 1.48-fold in100 mM of NaCl. In ‘Peto’, the relative *GR* gene expression in 25 mM of NaCl was equal to 2.28-fold and was decreased with an increase in salinity. In ‘Roma’, the least *GR* gene expression was observed in 25 mM NaCl compared to other cultivars (Fig. 6).

### Correlation matrix and relative expressions

**Fig. 7:**
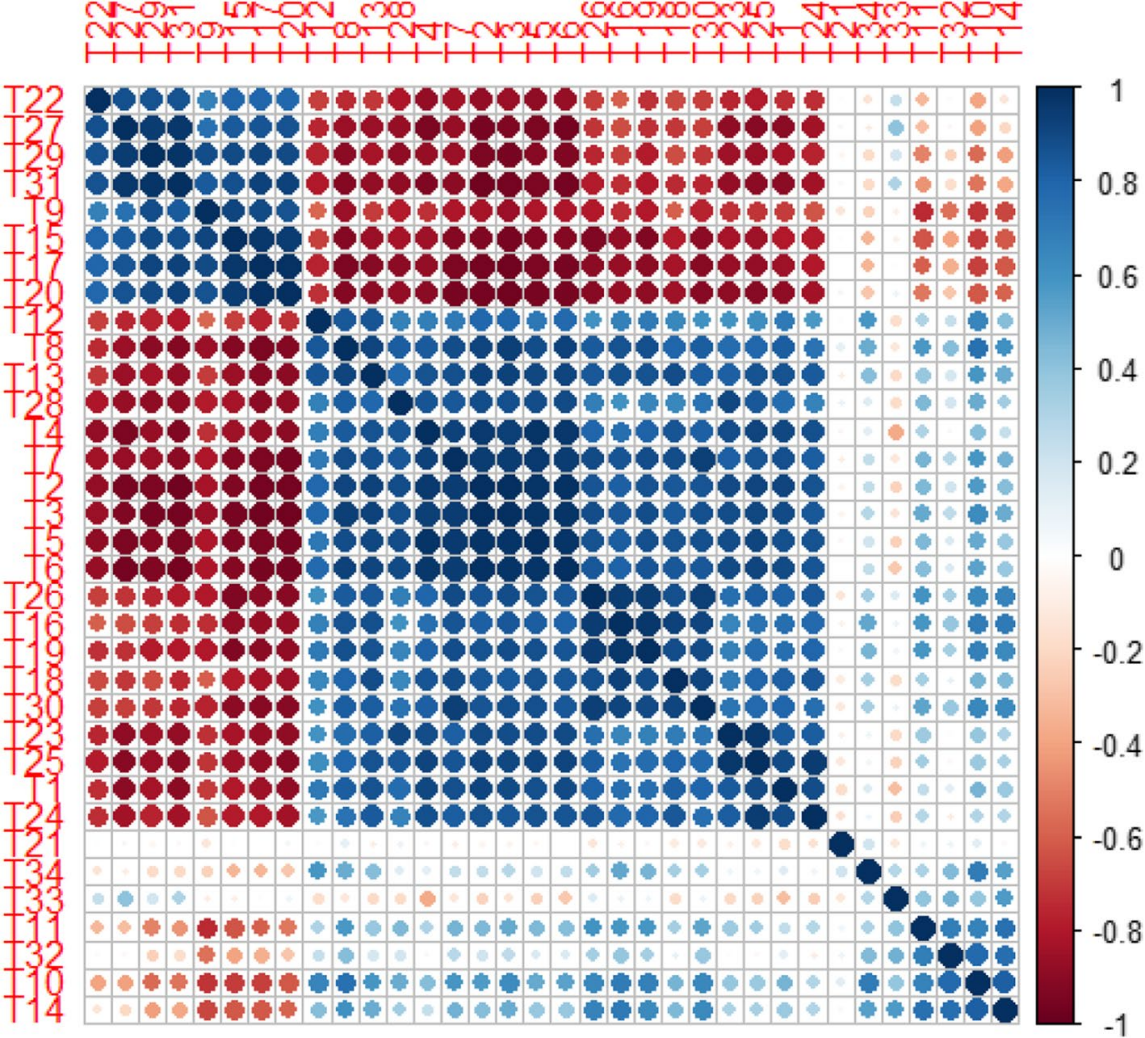
Heat map of Pearson’s correlation analysis. The studied attributes included T1 (germination%), T2 (Root FW), T3 (Root DW), T4 (Shoot FW), T5 (Shoot DW), T6 (Shoot length), T7 (Root length), T8 (Total soluble protein), T9 (H_2_O_2_), T10 (Catalase), T11 (Guaiacol peroxidase), T12 (Ascorbate peroxidase), T13 (Superoxide dismutase), T14 (Glutathione reductase), T15 (MDA), T16 (Ascorbate or AsA), T17 (Dehydroascorbate or DHA), T18 (AsA/DHA), T19 (Reduced glutathione or GSH), T20 (Oxidized glutathione or GSSG), T21 (GSH/GSSG), T22 (Proline), T23 (Chlorophyll a), T24 (Chlorophyll b), T25 (Total chlorophyll), T26 (Carotenoids), T27 (Na), T28 (K), T29 (Na^+^/K^+^), T30 (Ca), T31 (Electrolyte leakage), T32 (RQ*SOD*), T33 (RQ*APX*) and T34 (RQ*GR*)

The pearson’s correlation of morphological and physiological characteristics, elemental content and gene expression is presented in Fig. 7. The results were revealed two main groups in the evaluated traits that there is significant positive correlation within the groups and significant negative correlation between the groups. Group 1 included proline, Na, Na/K, EL, H_2_O_2_, MDA, DHA and GSSG, and group 2 of traits were APX special activity, protein, SOD, K content, shoot and root FW, root and shoot DW, root and shoot length, AsA, GSH, AsA/DHA, Ca and photosynthetic pigments content. Also, a positive correlation was observed among RQ*SOD*, RQ*APX* and RQ*GR* with SOD, APX and GR activity.

**Fig. 8:**
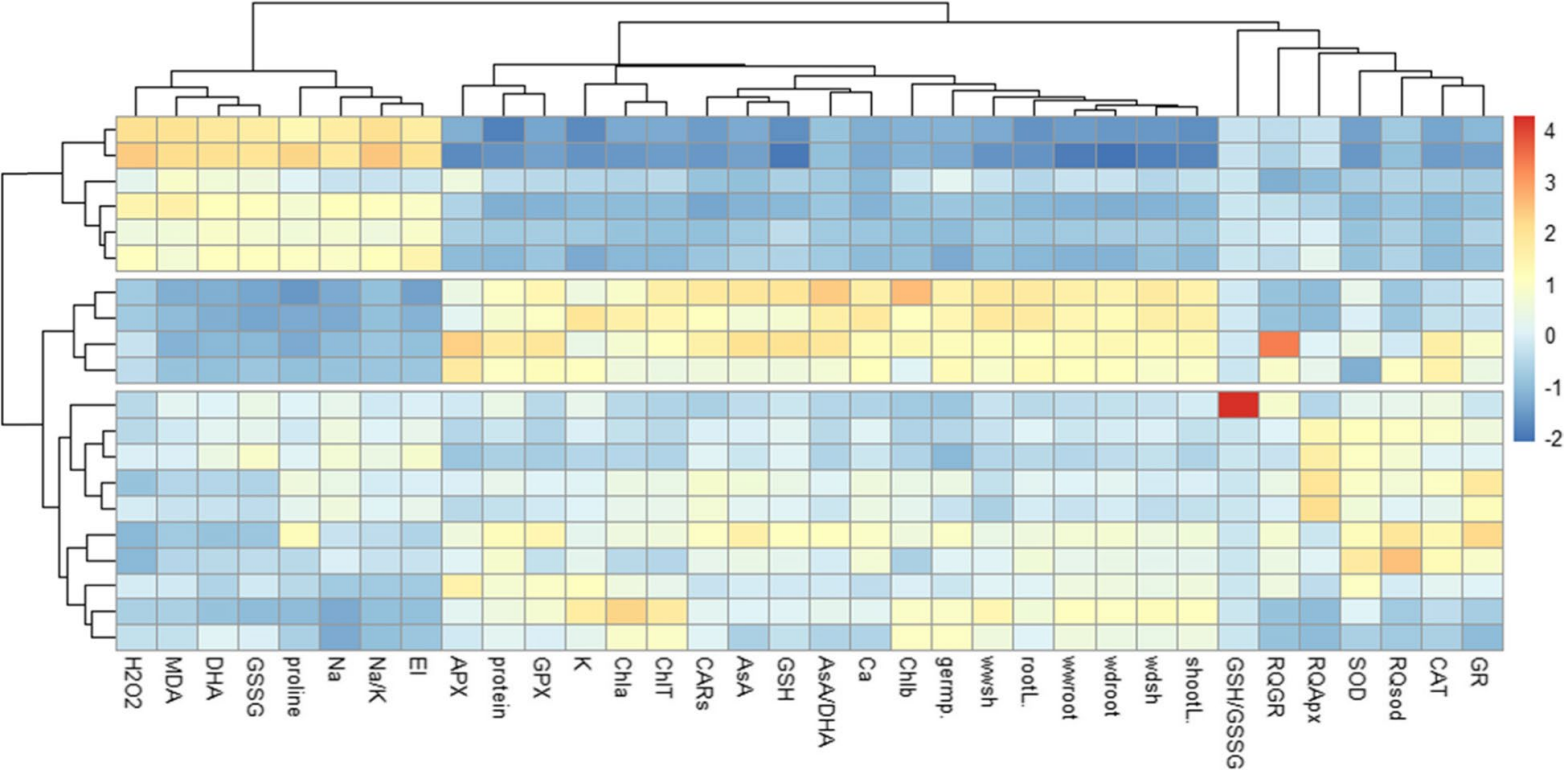
The morphological, physiological, biochemical and genes expression changes in four cultivars of tomato under salinity stress. Heat map representing of H_2_O_2_, MDA (Malondialdehyde), DHA (Dehydroascorbate), GSSG (Oxidized glutathione), proline, Na, Na^+^/K^+^, El (Electrolyte leakage), APX (Ascorbate peroxidase), protein, GPX (Guaiacol peroxidase), K (potassium), Chl a (Chlorophyll a), ChlT (Total chlorophyll), CARs (Carotenoids), AsA (Ascorbate), GSH (Reduced glutathione), AsA/DHA, Ca (Calcium), Chl b (Chlorophyll b), germination%, wwsh (Shoot fresh weight), RootL (Root Length), wdsh (Shoot dry weight), ShootL (Shoot length), GSH/GSSG, RQ*GR*, RQ*APX*, SOD (Superoxide dismutase activity), RQ*SOD*, CAT (Catalase) and GR (Glutathione reductase) responses in the cultivars under 0, 25, 50, 75 and 100 mM NaCl treatment

Heat map (Fig. 8) based on the response of tomato cultivars morphological, biochemical and nutrient elements content traits as well as gene expression under various salinity stress showed different responses in all cultivars. MDA, proline, DHA, GSSG, Na, Na^+^/K^+^, EL and H_2_O_2_ contents were increased under salinity stress. Moreover, the other traits were decreased under salinity stress. The results of heat map showed that the cultivars response to salinity stress was different so that, ‘PS-10’ and then ‘Peto’ were more tolerance than the other two cultivars, especially under the moderate salinity levels.

Cluster analysis and dendrograms in heat map (Fig. 8) showed two main groups in the evaluated traits of the cultivars under different salinity levels. Group 1 contained MDA, proline, DHA, GSSG, Na, Na^+^/K^+^, EL and H_2_O_2_ and group 2 contained other traits including shoot and root fresh weight, shoot and root dry weight, shoot length, APX, CAT, SOD, GR, AsA, GSH, DHA, GSSG, Chla, Chlb, Chl total, and the relative expression of *SOD*, *APX*, and *GR* genes. In general, cluster analysis of heat map for cultivars under salinity stress showed three main groups. Group 1 contained ‘PS-10’ and ‘Peto’ under 0, 25 and 50 mM NaCl, group 2 contained ‘Nora’ at 0, 25 mM NaCl, ‘Roma’ at 0 mM and ‘Peto’ under 75 mM NaCl and group 3 included ‘Roma’ under 25, 50, 75, 100 mM, ‘PS-10’ and ‘Peto’ at 100 mM, and ‘Nora’ under 50, 75 and 100 mM NaCl treatment.

#### Callus induction and regeneration under salinity stress with zinc oxide and iron nanoparticles

**Fig. 9:**
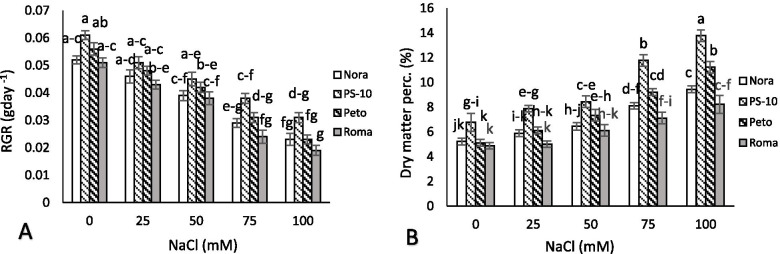
The effects of NaCl treatment on relative growth rate (RGR) (**A**) and dry matter percentage (**B**) of four tomato cultivars in *in vitro* condition. Means followed by the same letter on columns are not significantly different at 0.05 level, according to the Duncan’s multiple range test. Data are mean ± SD (*n* = 4 replicates)

Relative growth rate, dry matter percentage, osmotic potential, proline content, callus formation, and shoot formation under stress, and zinc oxide and iron nanoparticle treatment were analyzed in four tomatoes (*Solanum lycopersicon L.*) cultivars *in vitro* (Table 3). In the control, callus extract showed the least osmotic potential in all cultivars compared to stress conditions. An increase in NaCl amount in the medium increased the osmotic potential of the extract. The least osmotic potential was observed in ‘PS-10’ in 0 mM of NaCl and iron oxide, and zinc oxide nanoparticles (respectively −0.19, −0.2, and − 0.2 Mpa). In 100 mM NaCl, the most proline content was observed compared to control conditions. In 100 mM NaCl, least proline content was observed in ‘PS-10’ (8.2 fold) and the most content was observed in ‘Roma’ (16.24 fold). The zinc oxide and iron oxide nanoparticles with and without salinity stress decreased proline content in all cultivars. The most callus formation was observed with using iron oxide nanoparticles without salinity stress in ‘PS-10’ cultivar, and the least callus formation (6.33% in 100 mM NaCl) was observed in ‘Roma’. Using zinc oxide and iron oxide nanoparticles in control conditions and salinity stress increased callus formation; although no significant difference was observed between the two nanoparticles in terms of callus formation percentage. The highest shoot formation per explant with 12.5 shoots was associated with zinc oxide nanoparticles without salinity stress in ‘PS-10’, and the least shoot formation was associated with ‘Roma’ in 100 mM NaCl. The treatment of zinc oxide and iron oxide nanoparticles *in vitro* under salinity stress increased shoot regeneration to explant ratio; although no significant difference was observed between iron oxide and zinc oxide nanoparticles in terms of shoot regeneration (Table 2). With the increase in salinity stress, relative growth ratio was significantly decreased compared to control conditions in four cultivars. Hence, the most relative growth ratio was observed in ‘PS-10’ cultivar without salinity stress (0.061), and the least relative callus growth was observed in ‘Roma’ under 100 mM NaCl (0.019) (Fig. 9A). The dry matter percentage of explants was increased with an increase in salinity stress compared to control conditions. The highest dry matter content in 100 mM NaCl was observed in ‘PS-10’ cultivar (13.78%) and the least content was observed in the control sample, and in 25 mM of NaCl in the ‘Roma’ cultivar, respectively (4.88 and 5%) (Fig. 9B).

**Table 2:** The effects of NaCl treatment and application Fe_2_O_3_ and ZnO nanoparticles on osmotic potential, proline, callus formation and shoot formation of four tomato cultivars *in vitro* condition. Means followed by the same letter on columns are not significantly different at 0.05 level, according to the Duncan’s multiple range test. Data are mean ± SD (*n* = 4 replicates)

Cultivar	NaCl (mM)	Nano particle	osmotic potential (MPa)	Proline (μmolg^−1^FW)	Callus formation (%)	Shoot formation (Shoot/explant)
‘Nora’	0	0	−0.28 ± 0.047^a-d^	29 ± 0.94^q-w^	94.33 ± 0.11^a-c^	8.9 ± 0.14^fg^
		Zn	−0.27 ± 0.027^a-d^	23 ± 0.72^s-w^	96.26 ± 0.24^ab^	9.2 ± 0.12^f^
		Fe	−0.28 ± 0.094^a-d^	25 ± 0.88^r-w^	96.36 ± 0.46^ab^	9.1 ± 0.09^fg^
	25	0	−0.52 ± 0.058^d-h^	43 ± 0.17^o-s^	62.67 ± 0.21^f-k^	6.9 ± 0.08^mn^
		Zn	−0.49 ± 0.072^d-g^	36 ± 1.17^p-v^	64.91 ± 0.34^e-j^	8.1 ± 0.18^ij^
		Fe	−0.49 ± 0.11^d-g^	39 ± 1.12^o-u^	64.26 ± 0.38^f-k^	8.2 ± 0.09^h-j^
	50	0	−0.86 ± 0.045^j-o^	101 ± 0.98^n^	50.37 ± 0.19^j-q^	5.16 ± 0.19^q^
		Zn	−0.68 ± 0.14^g-k^	84 ± 1.14^n^	53.04 ± 0.27^i-o^	6.8 ± 0.08^mn^
		Fe	−0.78 ± 0.18^i-n^	89 ± 1.54^n^	52.32 ± 0.19^i-p^	6.8 ± 0.09^mn^
	75	0	−1.03 ± 0.12^m-s^	267 ± 1.11^f^	32.67 ± 0.18^r-v^	3 ± 0.014^s^
		Zn	−0.88 ± 0.072^j-p^	192 ± 1.14^ij^	37.21 ± 0.23^p-u^	3.6 ± 0.05^r^
		Fe	−0.92 ± 0.23^k-q^	203 ± 1.57^hi^	35.58 ± 0.34^q-v^	3.6 ± 0.04^r^
	100	0	−1.37 ± 0.14^uv^	381 ± 1.82^b^	13.67 ± 0.38^x-z^	0.8 ± 0.21^wx^
		Zn	−1.13 ± 0.027^p-t^	247 ± 1.14^g^	20.18 ± 0.41^v-z^	1.5 ± 0.08^u^
		Fe	−1.28 ± 0.047^tu^	269 ± 1.19^f^	16.17 ± 0.23^w-z^	1.7 ± 0.07^u^
‘PS-10’	0	0	−0.2 ± 0.094^a^	9 ± 0.72^w^	98.36 ± 0.32^a^	12.1 ± 0.1^a^
		Zn	−0.2 ± 0.14^a^	8 ± 0.86^w^	99.31 ± 0.18^a^	12.5 ± 0.05^a^
		Fe	−0.19 ± 0.12^a^	8 ± 0.98^w^	99.35 ± 0.98^a^	12.2 ± 0.24^a^
	25	0	−0.39 ± 0.11^a-f^	19 ± 0.94^u-w^	80.33 ± 0.37^c-e^	10.1 ± 0.2^d^
		Zn	−0.36 ± 0.072^a-e^	14 ± 0.72^vw^	83.41 ± 0.66^a-d^	10.8 ± 0.18^c^
		Fe	−0.37 ± 0.24^a-e^	16 ± 1.14^vw^	83.28 ± 0.94^b-d^	10.8 ± 0.09^c^
	50	0	−0.63 ± 0.098^f-j^	54 ± 1.22^op^	68.67 ± 0.51^d-i^	8.1 ± 0.11^h-j^
		Zn	−0.46 ± 0.11^b-g^	44 ± 1.78^o-s^	73.26 ± 0.75^d-g^	9.2 ± 0.14^f^
		Fe	−0.52 ± 0.094^e-i^	49 ± 2.14^o-q^	72.18 ± 0.68^d-g^	9.2 ± 0.17^ef^
	75	0	−0.92 ± 0.23^k-q^	141 ± 1.14^m^	49.36 ± 0.63^k-q^	4.9 ± 0.19^q^
		Zn	−0.7 ± 0.21^g-l^	91 ± 0.98^n^	59.18 ± 1.04^f-l^	6.1 ± 0.23^p^
		Fe	−0.85 ± 0.18^j-o^	104 ± 1.48^n^	58.36 ± 0.99^g-m^	6.2 ± 0.24^op^
	100	0	−1.01 ± 0.17^n-s^	238 ± 1.76^g^	24.62 ± 0.65^u-y^	2.2 ± 0.18^t^
		Zn	−0.81 ± 0.072^i-n^	144 ± 1.14l^m^	34.18 ± 0.68^q-v^	4.8 ± 0.15^q^
		Fe	−0.92 ± 0.11^k-q^	162 ± 1.87^kl^	31.48 ± 0.54^r-w^	5.1 ± 0.12^q^
‘Peto’	0	0	−0.23 ± 0.047^a-c^	18 ± 1.12^u-w^	96.75 ± 0.36^ab^	9.9 ± 0.11^d^
		Zn	−0.23 ± 0.25^ab^	15 ± 0.98^vw^	98.47 ± 0.96^ab^	11.2 ± 0.05^bc^
		Fe	−0.22 ± 0.21ab	16 ± 0.72^vw^	98.81 ± 0.24^ab^	11.3 ± 0.09^b^
	25	0	−0.49 ± 0.18^d-g^	20 ± 0.98^t-w^	70.13 ± 0.46^d-h^	8.6 ± 0.08^gh^
		Zn	−0.48 ± 0.072^c-g^	17 ± 1.14^u-w^	74.11 ± 0.35^d-f^	9.8 ± 0.04^d^
		Fe	−0.47 ± 0.094^b-g^	18 ± 1.74^u-w^	74.48 ± 0.39^d-f^	9.7 ± 0.09^de^
	50	0	−0.80 ± 0.072^i-n^	60 ± 1.12^o^	62.58 ± 0.24^f-k^	6.8 ± 0.11^mn^
		Zn	−0.57 ± 0.11^e-i^	46 ± 1.45^o-r^	66.31 ± 0.62^e-i^	8.2 ± 0.12^h-j^
		Fe	−0.71 ± 0.14^g-l^	48 ± 1.68^o-q^	66.27 ± 0.48^e-j^	8.4 ± 0.14^hi^
	75	0	−1.02 ± 0.12^n-s^	201 ± 0.98^hi^	40.71 ± 0.41^o-t^	3.2 ± 0.09^rs^
		Zn	−0.87 ± 0.18^j-o^	137 ± 172^m^	44.67 ± 0.54^l-r^	5.1 ± 0.05^q^
		Fe	−0.94 ± 0.12^l-r^	154 ± 1.65^lm^	42.12 ± 0.34^n-t^	5.2 ± 0.21^q^
	100	0	−1.17 ± 0.23^r-u^	288 ± 2.18^e^	20.58 ± 0.62^v-z^	1.3 ± 0.25^uv^
		Zn	−0.99 ± 0.21^m-s^	196 ± 1.45^ij^	26.18 ± 0.47^t-x^	2.8 ± 0.18^s^
		Fe	−1.08 ± 0.17^o-t^	208 ± 1.26^hi^	22.65 ± 0.58^u-y^	2.8 ± 0.14^s^
‘Roma’	0	0	−0.32 ± 0.072^a-e^	38 ± 0.98^o-u^	94.33 ± 0.42^a-c^	7.1 ± 0.25^lm^
		Zn	−0.34 ± 0.047^a-e^	30 ± 0.72^q-w^	95.83 ± 0.65^ab^	7.8 ± 0.41^jk^
		Fe	−0.33 ± 0.094^a-e^	32 ± 1.22^q-v^	95.21 ± 0.68^ab^	7.6 ± 0.17^kl^
	25	0	−0.78 ± 0.094^i-n^	50 ± 0.75^o-q^	54.33 ± 0.72^i-o^	5.1 ± 0.14^q^
		Zn	−0.76 ± 0.12^h-m^	39 ± 1.58^o-u^	57.22 ± 0.58^g-n^	6.6 ± 0.09^no^
		Fe	−0.78 ± 0.14^i-n^	42 ± 1.35^o-t^	56.32 ± 0.47^h-o^	6.6 ± 0.12^no^
	50	0	−1.19 ± 0.18^s-u^	179 ± 1.44^jk^	40.27 ± 0.58^o-t^	3.6 ± 0.18^r^
		Zn	−1.09 ± 0.16^o-t^	133 ± 1.28^m^	43.18 ± 0.78^m-s^	4.9 ± 0.24^q^
		Fe	−1.14 ± 0.21^q-u^	141 ± 1.78^m^	43.11 ± 0.75^m-s^	5.1 ± 0.28^q^
	75	0	−1.71 ± 0.18^wx^	311 ± 1.28^d^	23.36 ± 0.57^u-y^	1.2 ± 0.31^u-w^
		Zn	−1.32 ± 0.16^t-v^	203 ± 0.98^hi^	29.17 ± 0.12^s-x^	2.1 ± 0.26^u^
		Fe	−1.54 ± 0.14^vw^	219 ± 1.24^h^	27.21 ± 0.34^t-x^	2.3 ± 0.21^t^
	100	0	−1.85 ± 0.21^x^	471 ± 1.87^a^	6.33 ± 0.98^z^	0.2 ± 0.14^y^
		Zn	−1.52 ± 0.14^vw^	334 ± 0.98^c^	12.32 ± 0.58^x-z^	0.7 ± 0.27^x^
		Fe	−1.71 ± 0.23^wx^	351 ± 1.48^c^	9.08 ± 0.72^yz^	0.9 ± 0.22^v-x^

Mean with the same letter are not significantly different by Duncan grouping at (*P < 0.05*)

## Discussion

Salinity is one of the major stressors reduce the growth and productivity of plants. Salinity tolerance is a polygenic trait and, can be difficultly obtained using traditional breeding techniques (classic) under normal conditions *in vivo* [30]. The *in vitro* method is a fast and useful procedure for examination of salinity tolerance under controlled conditions [31].

Salinity tolerance during germination is depended on the germination speed and its ability against the effects of high salt concentration. However, low germination speed under salinity stress can be because of osmosis or ion effects of the salty medium. High salinity concentration can reduce osmosis potential and decrease the intake of water and nutrients needed for germination. Also, the salts or ions leave toxic effects on the embryo and affect germination. Salinity alters the activity of enzymes involved in nucleic acid and protein metabolism. With causing hormonal imbalances, salinity reduces the use of seed reserves thus declines seed germination [32]. The results of our experiments are consistent with the findings of Rahman et al. [33] showing that the delay in germination was in direct correlation with salt concentration, and reduction of seed hydration rate. In addition to salt concentration, salinity tolerance in plants is dependent on various genotypes of plants, types of salt, and medium osmosis potential [9]. Multiple authors have reported that salinity stress can affect the germination of seeds by reducing hydration. Also, the facilitation of ion absorption may change the activity of some enzymes and hormones inside the seed [34, 35]. Cell division and expansion, which is mandatory for growth and development, is strongly affected by salinity. Salinity reduces cell division in the first few minutes due to water loss by osmotic stress. After a few hours, the cells regain their original size, but the rate of expansion remains low, leading to a reduction in leaf, stem, and root growth rates [32]. Salinity controls photosynthesis by preventing the RuBisco activity, chlorophyll biosynthesis, and photosystems activity [12]. Chlorophyll concentration variations can be considered as a short-term reaction to stress. In salinity stress, chlorophyll is damaged because of an excessive increase in sodium content. The decreased chlorophyll content under salinity stress is because of magnesium deficiency. The decreased chlorophyll content is an indicator of growth damage on the plant [36]. In the present study, ‘PS-10’ cultivar could preserve an adequate amount of chlorophyll under salinity treatments. Carotenoids are active in photosynthesis as the light assistant receptors. The pigments absorb the blue light and protect chlorophyll against radiative oxidation. Also, carotenoids play role in the xanthophyll cycle and prevent the decomposition of chlorophyll [37]. Decreased chlorophyll concentration is probably because of the inhibitory effect of accumulated ions [9]. Decreased chlorophyll content in plants caused by salinity may be because of damage in chlorophyll molecule structure with increased chlorophyllase activity [38]. Sairam et al. [37] reported decreased chlorophyll content with increased salinity level in the salt-sensitive cultivars of wheat. They showed that chlorophyll reduction in sensitive cultivars was more than others. Decreased chlorophyll content as a result of salinity stress was also reported in cotton [39] and pumpkin [40], which are consistent with the findings of the present study. Increased salinity could decrease photosynthesis. This can be because of lower daily conduction, lack of complete induction of chloroplast, instability of pigments, destruction of chlorophyll structure, change in number, and combination of carotenoids, which can finally lead to a decreased dry weight [41].

Salinity stress disrupts Na^+^/K^+^ balance in cells with over accumulation of sodium and less accumulation of potassium. Also, intake of essential nutrients would be disrupted because of membrane damage and the competitive interactions of ions, which change in nutrient metabolism [42, 43]. The increase in extracellular Na^+^ concentration occurs due to the negative potential of the electrical charge in the plasma membrane (−140 mV), which leads to the inactive transfer of Na^+^ ions to the cytosol. Na^+^ ions limit the function of potassium, which acts as a cofactor in several reactions and therefore causes direct toxicity to the plant. In addition, Na^+^ appears to be detrimental to the structural and functional integrity of membranes [32, 44]. Over-absorption of sodium by the roots and transferring the ion to the shoots causes decreased absorption, transfer, and accumulation of other minerals like K, Ca, and Mg, and this can cause a lack of nutrients in plants [42]. Preservation of intercellular ion balance is one of the most vital mechanisms of salinity tolerance for plants. Ion balance is the sign of cell stability, and the dynamic prerequisite of the cell to control natural, biochemical, and physiological processes [43]. Balanced K, Mg, and Ca are vital for plant survival under salinity stress [12]. Salinity results in a lack of potassium in the plants, which can be explained by the competition between sodium and potassium in the same transfer system at the root level. Salinity stress decreases calcium content of leaves in three tomato lines, mainly because of the competition between sodium and calcium in terms of transfer from non-selective ion channels [45]. When sodium is accumulated in plants, it increases the toxicity effects in different physiological levels. The toxicity can not only cause nutrient dysfunction (K and Ca), but it can also destroy cells by creating osmosis stress [8, 46, 47].

In addition to ion and osmosis stresses, plants suffer from disruption of cell metabolism caused by oxidative damage [46]. Manai et al. [47] reported higher ROS levels as a result of increased MDA content in tomatoes under salinity stress. Salinity stress causes more activity of ROSs, which can disrupt the cellular membrane homogeneity in plants. It has been reported that salt treatment can increase lipid peroxidase in plant tissues [48]. This shows the inefficiency of the antioxidant system to prevent oxidative damage caused by salinity stress. Increased electrolyte leakage under various salinity levels can be a sign of explicit membrane damage [49]. An increase in electrolyte leakage has been reported as a result of increased salinity in wheat [37]. It was reported in an experiment that the increased level of total soluble sugar content, proline content, decreased MDA content, hydrogen peroxidation, and electrolyte leakage are useful indices of plants tolerance against environmental stresses. These properties can be used to select tolerant genotypes [50]. The present study showed that an increase in salinity stress could increase EL content significantly, which shows the accumulation of ROS under stress. Therefore, osmosis regulation of cell environment and ROS scavenging play a key role in reducing the membrane damage. These factors are dependent on the cultivar and stress intensity [51]. Under salinity stress, compatible solutes are accumulated to preserve the ions balance in cytoplasm and vacuoles. The main function of osmolytes is to protect cell structure and maintain osmotic balance through the constant flow of water [32]. Proline accumulation is associated with the conversion of Pyrroline-5-carboxylic to proline with the activity of Pyrroline-5-carboxylic reductase enzyme (P5CR) [52]. Proline preserves the macromolecule structure, especially enzymes, through the preservation of hydration capacity in the cell cytoplasm [53]. Intracellular proline promotes salinity stress tolerance and acts as an organic nitrogen storage during stress recovery. Proline stimulates the expression of salt-responsive proteins. As an antioxidant, it inhibits ROS, protects the photosynthetic apparatus thus promoting plant adaptation [54]. The effects of salinity stress on the accumulation of soluble sugars are associated with plants’ capability for salinity tolerance [30]. Furthermore, it has been reported that total protein biosynthesis was decreased under the effect of stresses such as drought or salinity [55]. Under stress conditions, the heat shock proteins (HSPs) act as chaperon molecules and prevent the accumulation of proteins. With the preservation of cell homeostasis, the HSPs increase stressors tolerance in plants. However, their function may vary under stress-free conditions [56].

The previous studies have shown that there is a significant correlation between ROS and response to abiotic stresses in plants. The ROS metabolism and antioxidants defense system were analyzed in four tomato cultivars *in vitro* under salinity [57]. Among the ROSs, H_2_O_2_ acts as the most underlying burst signal of oxidative stress because of high permeability in the membrane, and almost the long half-life time. An increase in H_2_O_2_ level was reported as a result of salinity stress in various plant genotypes. In tomatoes, a considerable increase in H_2_O_2_ was observed, especially in sensitive cultivars under salinity stress [48]. SOD activity is an underlying indicator to identify the sensitivity of plants because, this is the early defense line against ROSs [43]. Gomez et al. [58] have observed an increase in isozymes of pea SOD under salinity stress. Multiple studies have shown that SOD activity has been increased in types of tolerant pea and tomato [48, 58]. Decreased SOD activity was mostly observed in NaCl-sensitive plants; although it was increased in those NaCl-tolerant plants [58]. H_2_O_2_ produced by SOD is a strong oxidative factor. Therefore, the increased CAT and APX activity is a comparative feature, which helps to overcome the metabolism damage by reducing the toxic level of H_2_O_2_ [53, 58]. The elevated activity of CAT is associated with the increased tolerance against salinity stress due to the detoxification of H_2_O_2_ [59]. Changes in CAT activity are dependent on the growth and metabolic status of the plant, and the time and intensity of stress [9]. A major detoxification system of H_2_O_2_ in plant cells under abiotic stresses is the glutathione-ascorbate cycle, in which the ascorbate peroxidase isoenzyme (APX) plays a key role in the catalysis of converting H_2_O_2_ in to H_2_O [60]. APX plays a vital action in the ROS inhibition system and plants tolerance against salinity and alkaline stress. H_2_O_2_ detoxification in various cell parts is involved in AsA homeostasis and, simulates a balance in the ROS intercellular messenger network [61]. Ascorbate plays a major role in the intercellular regulation of H_2_O_2_ in plants [48]. GPX activity varies depending on the cultivar. In the tolerant cultivars against salinity and abiotic stresses, GPX content is more than sensitive cultivars. Such an increase in GPX activity in the tolerant cultivars shows the dominance of this enzyme on the surplus production of hydrogen peroxide [62]. GR increases the glutathione oxide conversion to the reduced glutathione using NADPH in the glutathione-ascorbate cycle. Increased GR activity has preserved the GSH/GSH + GSSG ratio at a desirable level, and has affected the efficiency of cell defense mechanisms to meliorate the tolerance of plants against environmental stresses [8]. Manai et al. [47] reported that GR activity was increased under salinity stress in tomatoes. Increased GR activity was observed after the recovery of treated plants under salinity stress. This could ascribe the GSH and GSSG content in plant tolerance. Several helicase proteins (such as DESD-box helicase and OsSUV3 dual helicase) have been reported to improve salinity tolerance by improving and maintaining photosynthesis and antioxidant machinery. Many studies have found differences in the expression or activity of antioxidant enzymes. These differences are associated with more tolerant genotypes and sometimes with more sensitive genotypes. Differences in the antioxidants activity may be due to the genotypic differences in the degree of stomatal closure or other responses that alter the rate of CO_2_ fixation [63]. Three main characteristics help plants adapt to salinity stress: ion removal, tissue tolerance, and salinity tolerance. Antioxidants seem to play a role in tissue tolerance under salinity [54]. Ascorbate is the most frequent antioxidant in plant cells, which is present in all intracellular organs, and apoplast space. Ascorbate can show a direct reaction with hydroxyl radicals, superoxide, and single oxygen, and can also revive the oxidated forms of alpha-tocopherol [4]. Ascorbate plays a vital role in the AsA-GSH cycle to control the ROSs by its capacity to donate an electron and create two-form stability [8]. Non-enzyme antioxidants such as AsA and GSH, alpha-tocopherol, and flavonoids are constantly in interaction with antioxidant enzymes such as POX, CAT, SOD, APX, GPX, GR, and GST to prevent overproduction of ROSs [64]. The AsA-GSH cycle or Asada-Halliwell cycle in plant cells is the main antioxidant defense route for detoxification of H_2_O_2_, which is formed of isoenzyme antioxidants of AsA and GSH, and DHAR, MDHAR, APX, and GR [10, 65]. In the AsA-GSH cycle, H_2_O_2_ is destroyed by direct interference of AsA and indirect interference of GSH. As a result, both AsA/DHA and GSH/GSSG ratios were decreased under salinity stress. Salinity stress increased the GR activity and decreased GSH content, which shows the overuse of GSH [12]. Manai et al. [47] reported increased GR activity in tomatoes treated with NaCl. In the early salinity levels, AsA content was increased in the tolerant cultivars and was then decreased with the intensified stress. Increased activity of DHAR guarantees the efficiency of AsA. This can remove a higher level of H_2_O_2_ under stress conditions. The GSSG produced while the regeneration of AsA is revived by DHAR and by GR dependent on NADPH to GSH to allow inhibition chain reactions of H_2_O_2_ are completed and continued by APX [48]. AsA significantly scavenges free radicals, thus reducing oxidative stress damage, further helps protect membranes and also acts as an adjuvant of violaxanthin de epoxidase, resulting in supports extra energy dissipation stimulation. In addition to increasing the activities of CAT, POD and SOD; AsA increases the growth of plants under salt stress. GSH protects proteins against denaturation as a substrate for glutathione peroxidase (GPX) and glutathione-s transferases (GST), which is regulated by ROS deletion [32, 54].

The interference of antioxidant defense to enhance salinity tolerance of plant genotypes may be reflected in the gene transcription level. The transcription studies in plants show the correlation of salinity stress response and other environmental stresses with gene expression [15]. In Arabidopsis plant, different degree of *SOD* gene expression was observed under salinity. The type of expressed *SOD* genes varies depending on the type of stress. In some types of stress, the majority of chloroplast genes (*FeSOD*) such as *FSD1,2* and *CU/Zn SOD* including cytoplasmic *CSD1* and chloroplast *CSD2* can be induced. Also, *SOD* expression regulation shows no special pattern for the ecotypes. Different tolerance and the response of every genotype of *Arabidopsis* is dependent on stress factors [13]. *SOD* genes act as the early defense line against oxidative stress in plants [15]. Also, it has been reported that the expression of the majority of *SOD* genes results in the improvement of oxidative stress tolerance in plants [66]. Up-regulation of *SOD* under stress conditions same as other antioxidant genes can act as an effective defense line to inhibit H_2_O_2_ [15]. APX as the main element of the ascorbate-glutathione cycle plays a key role in the balance of intercellular ROS accumulation. As a general principle, reset of this gene improved stress tolerance in these plants [66]. The studies have reported that salinity can increase the transcription level of APX1, 4, and 6 in rice leaves. Although *APX2* gene expression has not been changed, *APX8* has been decreased a little [67]. In sorghum, the *GPX/APXs* genes were mostly regulated in leaves; although they were less regulated in root tissues [68]. Under salinity, genotypes of *APX* isoforms showed their expression based on types of tissue and stress time [69]. Reddy et al. [70] reported the reverse correlations between transcription level and relevant enzyme products. The difference between gene expression and enzyme activity in different cultivars showed that enzyme changes were not created at mRNA level, but also it was regulated at the post-transcription level. This may be because of activation/inactivation or synthesis/decomposition of enzyme activity caused by salinity. In this study, *GR* gene expression was increased with the beginning of salinity stress and was then decreased with an increase in salinity levels. Such an increase in ‘PS-10’ cultivar was more than other cultivars. Other studies have revealed that the cytosolic, chloroplast, and mitochondrial transcript levels of GR in rice seeds were increased under salinity stress and with exterior H_2_O_2_ treatments [71]. In pea, the cytosolic *GR* was induced in NaCl-tolerant varieties; although it was not expressed in NaCl-sensitive varieties under salinity stress [72]. Salinity and other abiotic stresses induce metabolic imbalances that lead to the production of ROS. Thus, plant root or branch proteomics show the expression of ROS inhibitory proteins such as SOD, CAT, GPX, APX and GR [44, 63].

Adding salt to the medium decreases the osmosis potential of the medium caused by salinity stress, which left a negative effect on callus induction and regeneration of tomato cultivars. Watanabe et al. [73] showed that *in vitro* culture is an acceptable method to study the salinity tolerance in many plants. The results could be generalized to *ex vitro* conditions. Multiple authors have reported using NaCl for salinity screening *in vitro* on different plants [74]. Over the years, considerable improvement was observed in salinity tolerance in some types of plant products using nanotechnology [75]. The application of nano-micronutrients has been reported to reduce the harmful effects of salinity on plants (for example, by improving water relations, photosynthesis and nutrition and regulating antioxidant defenses and increasing osmotic and amino acid protection levels [76]. *In vitro* cultivation provides a controlled and homogenous environment for analysis of physiological processes of plants, especially at the cellular level under various treatments of chemicals [77]. Callus induction increased the dry matter percentage in presence of NaCl concentration and decreased RGR in all tomato cultivars [27]. The callus of growing tomato showed higher dry weight in presence of NaCl compared to control conditions [2]. Zinc oxide and iron oxide nanoparticles decreased the effects of salt by reducing the osmosis potential of calluses in the medium. Osmosis regulation in callus is obtained by the accumulation of Na and Cl. Similar results were also reported for wheat genotype callus [78]. Sotiropoulos et al. [79] reported that the explants under salinity stress *in vitro* encounter increased osmosis potential of the medium as a result of increased osmosis potential of explants with high sodium content and toxic effects of sodium. The findings of Mozafari et al. [23] on grape *in vitro* condition showed that using iron nanoparticles could decrease the destructive effects of salinity on the explants. Sodium toxicity can reduce the salt content using iron chelate in grape. Also, sodium and chlorine were decreased, and the potassium content was increased under salinity stress using iron nanoparticles. In this case, iron can be absorbed more rapidly. Iron nanoparticles have great potential for improving salinity stress in plants [80], but information on the specific metabolic pathways (at the molecular level) that they regulate is not fully understood [81].

An increase in NaCl content could increase proline content in all cultivars. The accumulation varied dependent on the cultivars and NaCl level. The results were consistent with the findings of Emilio et al. [82] on *L. esculentum* and *Lycopersicon pennellii*. Martinez et al. [83] reported a positive correlation between proline accumulation and NaCl tolerance in potatoes. Under salinity stress, a higher level of glutamate was consumed as the chlorophyll and proline pre-product for proline production. Proline can regulate the osmosis pressure of cells under various stresses. Also, released proline can destroy ROSs produced by stresses [84].

Mercado et al. [85] examined tomatoes using tissue culture techniques to select *in vitro* salinity tolerance. The results of some researchers showed that NaCl results in a significant decrease in callus induction. The reduction of callus related traits is because of hyperosmotic stress, which decreases the availability of water. As a result, cell growth is decreased and cell division is stopped. Hence, the CFW, CRGR, CWC, and CSP are decreased in calli under salinity stress compared to control ones. Such reduction helps the plant to save energy for defensive goals [22, 86]. The results of the present study showed that using ZnO-NP and Fe_2_O_3_-NP can decrease harmful salinity effects. Hence, sufficient Zn content can reduce sodium accumulation and help the plant tolerate salinity [29]. Zinc (Zn) plays a key role in controlling the production and detoxification of ROSs, which can damage the membrane lipids and sulfhydryl groups [27]. Also, it may help restricting lipid peroxidation, because this is a protective component stabilizing the biologic membranes against ROS [87]. Farouk et al. [88] reported that the use of Zn-NPs under salinity reduced the harmful effects of salt through osmolytes biosynthesis and ion regulation. ZnO nanoparticles increase the chlorophyll content in the plant and affect photosynthetic systems under salinity stress (for example, by the activity of carboxylase ribulose-1, 5-bisphosphate). Zinc nanoparticles can improve plant growth by affecting the electron transfer chain and increasing enzymatic antioxidants. Zinc nanoparticles on plants under salinity stress also reduced ion leakage and improved the Hill reaction [89]. The iron function in oxidation reactions of electron redox has changed it into an underlying element with a basic roles in major biochemical reactions such as oxygen exchange, intense reaction with free radicals, DNA synthesis, electron exchange in chloroplasts and mitochondria, and total energy production [90] Iron is involved in many vital cellular processes, including chlorophyll biosynthesis, respiration, and photosynthesis [76]. Rawat et al. [91] found that iron-NPs increase gene expression in small and large subunits of enzymes involved in photosynthesis and thus enhance the process. Iron and zinc stimulate the antioxidant enzymes activity in plants, and help reduction of the effects of free radicals [90]. As iron and zinc play role in nitrogen metabolism and protein structure, using them can increase total protein content as well [84].

## Conclusion

Our results showed that salinity stress in the culture medium limited germination and seedling growth of four tomato cultivars. With increasing salinity level up to 100 mM, hydrogen peroxide, MDA content and electrolyte leakage in tomato seedling increased. With increasing salinity, accumulation of sodium was increased and, a decrease in calcium and potassium content was observed. The activity of CAT, APX, GPX and GR antioxidant enzymes and AsA and GSH antioxidants increased at low and medium salt levels and, otherwise decreased with increasing salinity. The relative expression of *APX* genes increased at all levels of salinity, and *SOD* and *GR* activities were added up in low and medium salt conditions. The use of iron oxide and zinc oxide nanoparticles significantly increased the percentage of callus formation and regeneration of explants by reducing the osmotic pressure of the environment. ‘PS-10’cultivar showed reasonable growth potential in all salt concentrations considering the activities of antioxidant enzymes and gene expression levels. Therefore, it can be concluded that ‘PS-10’ cultivar can be a potential salinity tolerant cultivar for the future use in breeding programs. However, further studies are required for a better understanding of signaling pathways and the expression of genes involved in these pathways. Also, it is necessary to determine the function of nutrient nanoparticles in response to stress in plants to improve the ability of tomato cultivars to be tolerant against the stresses in a wide range of environments.

## Methods

### Plant material, treatments and culture conditions

Tomato seeds of four cultivars (‘Nora’, ‘PS-10’, ‘Peto’, and ‘Roma’), which were provided from Pakan Bazr Company, Isfahan, Iran, were used in this study. The seeds were surface sterilized with 70% ethanol for 1 min and then with sodium hypochlorite (2%) for 10 min and thoroughly washed with sterile distilled water for three times. Then, seeds kept for germination in a ½ MS supplementing with 0, 25, 50, 75 and 100 mM NaCl to the media and incubated under 16 h illuminations (70 μmolm^−2^ s^−1^) and 28 °C temperature for 3 weeks. Then, germination test, electrolyte leakage, Na, K, and Ca concentration, chlorophylls, total antioxidant activity and antioxidant enzymes (SOD, APX, CAT), Malodialdehyde (MDA), H_2_O_2_, total soluble protein and expression of genes (*APX*, *SOD* and *GR*) were performed.

14-day-old seedlings were used as explants. For callus induction hypocotyl explants were placed in MS medium supplemented with 1 mgL^−1^ 2,4-D and 1 mgL^−1^ BA and for organogenesis, cotyledonary nodes were placed on shoot induction media (MS media supplemented with 2 mgL^−1^ BA and 0.5 mgL^−1^ IAA). All the cultures were maintained at 25 ± 1 °C under 16 h illumination (70 μmolm^−2^ s^−1^). The explants were cultured in MS media supplemented with NaCl treatments (0, 25, 50, 75 and 100 mM) and nanoparticles of Fe_3_O_4_ (3 mgL^−1^), ZnO (30 mgL^−1^). The cultures were kept for six weeks to study their growth potential and regeneration capacity. After six weeks, the samples were analyzed for their relative growth rate (RGR), dry matter percentage (DM) and osmotic potential (ψ_s_).

### Determination of seed germination characteristic

Germination characteristics of seeds culture *in vitro* under NaCl treatments were evaluated after 3 weeks. Traits such as germination percentage, germination rate, shoot length, root length, shoot and root fresh and dry weight were evaluated.

### Antioxidant enzymes assay

For the extraction of guaiacol peroxidase (GPX), CAT, SOD, GR and total soluble proteins, 0.2 g of the plant tissue was homogenized in liquid nitrogen. 2 ml phosphate buffer (pH = 7.5) containing EDTA (0.5 M) was added. The samples were incubated at 4 °C for 15 min and were centrifuged at 15 rpm [30]. Due to the instability and very low half-life of ascorbate peroxidase with *ex-vivo* conditions and for the keeping structure of the compound, we tried to use polyvinylpyrrolidone 5% and ascorbate (2 ml) to the respected enzyme solution [92]. Guaiacol peroxidase (GPX) activity, based on the amount of tetraguaiacol, was obtained using an extinction coefficient of 26/6 m*μ* cm^−1^ [80]. For catalase (CAT) activity essay, the reaction mixture was containing 1.5 ml phosphate buffer (100 mM, pH = 7), 0.5 ml H_2_O_2_ (7.5 mM) and 50 μL of extracted enzyme solution. The absorbance at 240 nm during 1 min was measured [93]. Super oxide dismutase (SOD) activity was evaluated in a reaction mixture with the addition of 0.1 ml riboflavin (60 μL) and the samples absorbance was recorded at 560 nm [94]. Glutathione reductase (GR) activity assay was determined in a reaction mixture with the addition of glutathione oxide (2 mM) and the absorbance was recorded at 412 nm per minute [37]. Ascorbate peroxidase (APX) activity was calculated using the extinction coefficient of 2.8 mmol^−1^ cm^−1^. The resulting number indicates the activity of ascorbate peroxidase based on micromoles of oxidized ascorbate per minute [95].

### Total Soluble Protein content

Reaction solution for total soluble protein content was contained 100 μL of enzyme solution, 200 μL of Bradford regent and 700 μL of deionizer water. 2 min after the complex formation. Bradford regent shows the highest integration with the amino acids. Absorbance was evaluated at 535 nm. Protein content of the samples was calculated based on standard curve obtained from the defined amounts of bovine serum albumin [96].

### Hydrogen Peroxide (H_2_O_2_) content

0.2 g of the plant material was homogenized in 2 ml of 0.1% tricloroacetic acid and centrifuged at 12000 g for 15 min. 0.5 ml supernatant was added to 0.5 ml of phosphate buffer (10 mM, pH = 7) and 1 ml of Iodide potassium (1 M). The samples absorbance was measured at 390 nm. Standard curves were established with the different concentrations of hydrogen peroxide [97].

### Malondialdehyde (MDA) content

0.2 g of the plant sample was homogenized in 2 ml of 20% Tricloroacetic acid containing 0.05% TBA. The samples later were incubated in 95 °C for 30 min and they were transferred to the ice. The samples were then centrifuge at 10000 rpm for 10 min and the absorbance was measured at 532 and 600 nm. The extent of lipid peroxidation was obtained from the difference between the absorption wavelengths in the extinction coefficient of 155 mmol cm^−1^ [98].

### Ascorbate activity

0.2 g of the plant sample was homogenized in 1 ml of metaphosphoric acid and centrifuged for 15 min at 25 °C and 22,000 rpm. To measure the reduced ascorbate, 150 μM of phosphate buffer (pH = 7.4) and 200 μl of distilled water were added to the resulting supernatant. The resulting mixture was vortexed and then 400 μl of 10% trichloroacetic acid, 400 μl of 44% phosphoric acid, 400 μl of 4% bipyridyl in 70% ethanol and 200 μl of 3% FeCl3 were added. After vortex, the samples were kept at 37 °C for one hour and then the absorbance of the samples was recorded at 525 nm. In the measurement of total ascorbate, 100 μl of 10 mM dithiotritol was added to the reaction mixture. Reduced ascorbate was used to draw the standard curve [99].

### Glutathione activity

0.2 g of the plant sample was homogenized in 2 ml of 5% sulfosalicylic acid solution and then centrifuged at 15,000 rpm for 10 min. 300 μl of the supernatant was neutralized by adding 18 μl of 7.5 M triethanolamine. 50 μl of the above samples with 700 μl of 0.3 mM NADPH, 100 μl of 5 and 5-dithiobis 2-nitrobenzoic acid (DTNB), 150 μl of 125 mM phosphate buffer (pH = 6.5) containing EDTA 6.3 mM was mixed and 0.1 unit of glutathione reductase enzyme was added to the sample and finally the adsorption of the samples was mixed in 412 mM and 0.1 unit of glutathione reductase enzyme was added to the sample and finally the adsorption of the sample was measured at 412 nm. Reduced glutathione and oxide were used to draw the standard curve [100].

### Proline content

Proline concentration was measured in fresh plant tissue by Bates et al. [101] method and the absorbance of the samples was measured at 520 nm wavelength using a spectrophotometer. The control solution contained pure toluene.

### Chlorophylls and carotenoids content

0.5 g of the leaf sample was transferred to a small test tube and 3 ml of the test tube solution was transferred to a cuvette and, absorption light was recorded at 480, 649 and 665 nm. Due to the sensitivity of chlorophylls to light, the experimental steps were performed in a semi-dark environment. The values of chlorophyll a, b and carotenoids were calculated based on the following equations [102].

### Electrolyte leakage

0.2 g of the plant sample in 30 ml of mannitol (0.4 M) was incubated in a 50 ml plastic container at 24 °C for 20 h on incubator shaking (80 rpm). Electrolytic conductivity was measured using a conductivity meter. The samples are left in an autoclave at 120 °C for 3 min and then cooled to room temperature and the equilibrium volume reaches 30 ml. Electrolyte leakage was then calculated [103].

### Elemental content

To measure the concentration of elements, the leaves were first harvested and placed at a temperature of 70 °C. In order to measure the concentration of elements, 1 g of the dry sample was placed in an oven at 550 °C for 4 h. After the samples had cooled, 10 ml of 2 N hydrochloric acid was added to the samples and the heater was gently heated until half of the acid had evaporated. The solution was collected from the passed filter paper and the extracted filters were collected in a 50 ml balloon. Then with distilled water, the final volume of extracts reached 50 ml. (The amount of sodium and calcium in the samples through the atomic absorption device Model AA200 made in Malaysia under the license of Pekin Elmer USA measured).

### Semi-quantitative and quantitative RT-PCR

#### Total RNA extraction and cDNA synthesis

In order to extract RNA of shoots from seedlings treated at different levels of NaCl and control in *in vitro* conditions; the kit RNx Plus (Cinagene Company) was used. For cDNA synthesis, one μl of RNA template (1 μg/ml) was mixed with 25 μl of 2X Reddy MixTM Master Mix, 1 μl primer (Table 1), 1 μl reverse transcriptase blend and DEPC treated water up to 50 μl. Reverse transcription and PCR amplification was performed using the following thermal conditions: First strand cDNA synthesis at 47 °C for 30 min (1 cycle), reverse transcriptase inactivation and initial denaturation at 94 °C for 2 min (1 cycle), denaturation at 94 °C for 20 s, annealing at 55 °C for 30 s, extension at 72 °C for 5 min (40 cycles) and final extension at 72 °C for 5 min (1 cycle).

### RT-PCR

Two-step RT-PCR conditions using Im Prom II reverse transcription kit for cDNA synthesis (Promega) with oligo dT primers were performed according to the instruction manual for increased sensitivity. The cDNA product was diluted 40 times and 1 μl was used as a template for PCR with a reaction mixture containing 5 mM of each primer, 2 mM dNTPs, 1X Taq buffer and 1 unit of Taq polymerase (BIOLINE) in a final volume of 20 μl. The DNA fragment was amplified for 35 cycles using the following thermal conditions: denaturing DNA template 94 °C for 30 s, primer annealing 5 °C below primer Tm for 15 s, DNA synthesis 72 °C for 1 min. The primer sequence (designed by Oligo Porgram) is shown in Table 3.

**Table 3:** Sequences of primers used for real-time PCR

Gene (accession number)	Direction	Sequences (5′ → 3′)
*SOD* (NM 001247840.2)	F	CAG AGG GTG CTG CTT TAC AA
	R	GGT CAC AAG AGG GTC CTG AT
*APX* (NM 001247702.2)	F	GCA GCT GCT GAA GGA GAA GT
	R	CAC TGG GGC CAC TCA CTA AT
*GR* (NM 001247314.2)	F	GCCAAAATCTGGATGATGCAC
	R	GATAAGCTACCAACAGAAGCAG
*Actin* (NM 001330119.1)	F	GCC CCT AGC AGC ATG AAG AT
	R	GCA CTT CCT GTG GAC AAT GG

### Synthesis of ZnO NPs

Nanoparticles of ZnO with a mean basic particle size of 30 nm were bought from Sigma-Aldrich Company, California, USA. To make various concentrations of ZnO-NP (30 mgL^−1^), 1.5 g of solid ZnO-NP was solved in 1 L distilled water, and in order to homogenization, a sonicator was applied to the solution at 100 W, 40 kHz for 30 min. A magnetic stirrer bars were used in the suspensions before application to prevent the particles aggregation. The nanoparticle suspensions were centrifuged (3000 xg for 1 h) and filtered (0.7-μm glass filter) before adding to culture media.

### Synthesis of Fe_3_O_4_ NPs

First, the silicone oil was poured into the crystallizer dish and placed on the heater. Then, the heater degree was set on 100 °C and a thermometer was put inside it to stabilize the bath temperature at 80 °C. A single-mouth balloon, containing 40 ml distilled water, was inserted into the ultrasonic and 4.83 g hexahedron chloride and 3.34 g heptahydrate Fe sulfate were added. Then, the balloon was placed in the silicone oil using a base and clamp and stirred by a magnet as much as possible. Once the salts were completely dissolved, 12 ml concentrated ammonia was added to the solutions immediately. Adding ammonia changed the color of the solution immediately to black. The balloon was closed with a cap or parafilm and stirred for up to two hours in this condition. The system was checked from time to time to control the temperature and agitation. Then, the temperature of the balloon was allowed to reach the ambient temperature and the contents of the balloon were separated from the reaction solution. Then, it was washed three times using a 1:1 solution of ethanol: water to remove the remaining reactants and was dried in an oven at 80 °C.

### Features of Synthesized Nano-sorbents

XRD and FTIR techniques were used to evaluate the synthesis accuracy and the features of Fe_3_O_4_ magnetic nanoparticles.

### XRD Spectrum

**Fig. 10:**
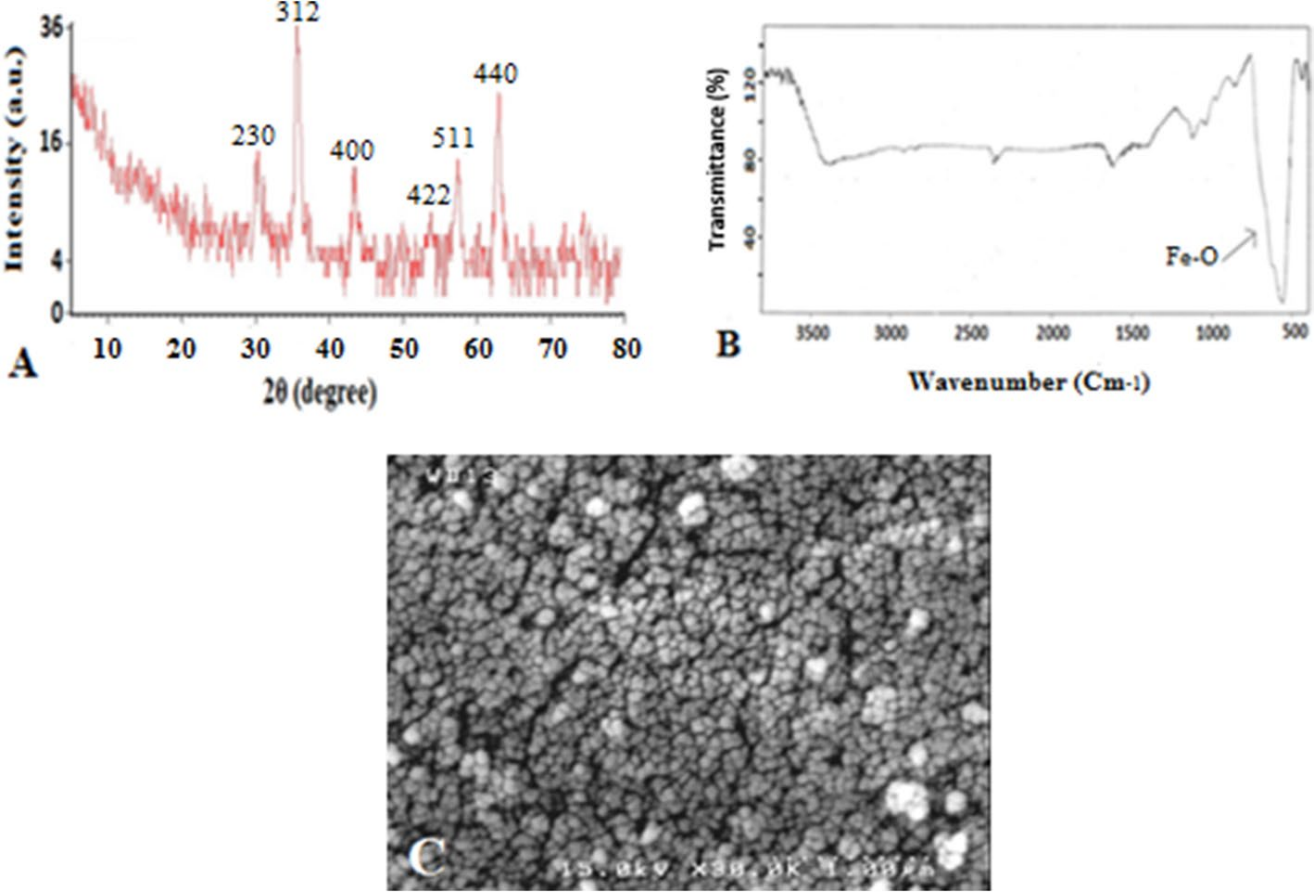
XRD spectrum of Fe_3_O_4_ nanoparticles (**A**), FT-IR spectrum of Fe_3_O_4_ nanoparticles (**B**, FE-SEM of Fe_3_O_4_ nanoparticles (**C**)

The XRD spectra of the synthesized nanoparticles are shown in Fig. 10 (A). The peaks formed in 2θs equal to °007/30, °782 / 53, °239 / 43, °601 / 35,007 / 30 °and 63 / 058 ° confirm the synthesis of Fe nanoparticles.

### FT-IR Spectrum

The infrared spectroscopy of the Fourier transform is often used to determine the nature as well as to confirm the presence of functional groups in the structure. Figure 10 (B) shows the FT-IR spectrum of Fe_3_O_4_ nanoparticles in the range of 500–4000 cm-1. In this figure, a peak is seen around 570 cm-1, related to the tensile vibrations of Fe-O which indicates that Fe-O particles were synthesized well.

### FE-SEM images

Scanning electron microscopy was used to investigate the morphological features of the synthesized nanoparticles. Figure 10 (C) shows the FE-SEM image of Fe_3_O_4_ Nanoparticles.

After culturing the explants, hypocotyl for callus production and nodes for shoot production in MS media and supplement with NaCl treatments (0, 25, 50, 75 and 100 mM) and Fe_3_O_4_ (3 mgL^−1^)**,** ZnO (30 mgL^−1^) callus formation percentage, callus PGR, callus DM, callus O.P. and shoot formation (shoot/explant) were measured.

$$\mathrm{Callus}\ \mathrm{RGR}=\left({\mathrm{FW}}_2-{\mathrm{FW}}_1\right)/\mathrm{Number}\ \mathrm{of}\ \mathrm{days}$$

FW_1_: The initial fresh weight.

FW_2_: The fresh weight at end of test period

$$\mathrm{Callus}\ \mathrm{DM}=\left({\mathrm{DW}}_2/{\mathrm{FW}}_2\right)\times 100$$

DW_2_: The dry weight at end of test period.

Osmotic potential was determined with an osmometer (030), using sap extracts from fresh calli tissues. Osmolarity was expressed as MPa using the formula ψ_s_ = 0.00227 k, where k = osmolarity in mosmol kg^−1^ [104].

The experiment was conducted as completely randomized design (CRD) with four replications. Data were subjected to analysis of variance and the means were separated using LSD at 5%.

## Data Availability

The data that support the findings of this study are available from the corresponding author upon reasonable request.

## Abbreviations

MS: Murashige and Skoog medium
RT-PCR: Reverse transcription-polymerase chain reaction
SOD: Superoxide dismutase
CAT: Catalase
APX: Ascorbate Peroxidase
MDA: Malondialdehyde
GPX: Guaiacol peroxidase
GR: Glutathione reductase
AsA: Ascorbate
DHA: Dehydroascorbate
GSH: Reduced Glutathione
GSSG: Oxidized Glutathione
